# Provider and patient perspectives on the diagnosis and treatment of Alzheimer's disease: A global perspective from the Global Alzheimer's Leadership Series (GoALS)

**DOI:** 10.1002/alz.71536

**Published:** 2026-06-15

**Authors:** Philippe Amouyel, Sandrine Andrieu, Angela Bradshaw, Ralph C. Carmona, Magali Dumont, Lea Tenenholz Grinberg, Takeshi Iwatsubo, Oskar Hansson, Clifford R Jack Jr, Gregory A Jicha, Simin Mahinrad, Eric McDade, Catherine J Mummery, Ronald C Petersen, Shelagh Robinson, Julie A. Schneider, Lenny Shellcross, Amanda G Smith, Heather M. Snyder, Beth Tapply, Charlotte Teunissen, Wiesje M. van der Flier, Bruno Vellas, David Wallon, Jeff D Williamson, Donna Wilcock, Maria C. Carrillo

**Affiliations:** ^1^ Foundation Alzheimer Paris France; ^2^ LabEx DISTALZ Inserm, Centre Hospital, University of Lille Lille France; ^3^ UMR1167, RID‐AGE Institut Pasteur de Lille Lille France; ^4^ Epidemiology and Public Health Department University of Lille Lille France; ^5^ IHU HealthAge Toulouse University Hospital Toulouse France; ^6^ Aging Research Team, Centre for Epidemiology and Research in Population Health (CERPOP), INSERM University of Toulouse Toulouse France; ^7^ Department of Epidemiology and Public Health Toulouse University Hospital Toulouse France; ^8^ Alzheimer Europe Senningerberg Luxembourg; ^9^ University of California Oakland California USA; ^10^ Southern Maine Community College South Portland Maine USA; ^11^ Fondation Alzheimer Paris France; ^12^ Department of Neurology University of California at San Francisco San Francisco California USA; ^13^ Department of Pathology University of California at San Francisco San Francisco California USA; ^14^ Department of Pathology University of Sao Paulo Sao Paulo Brazil; ^15^ Department of Neuropathology The University of Tokyo Tokyo Japan; ^16^ Clinical Memory Research Unit, Department of Clinical Sciences Malmö Faculty of Medicine Lund University Lund Sweden; ^17^ Department of Radiology Mayo Clinic Rochester Minnesota USA; ^18^ Sanders‐Brown Center on Aging University of Kentucky Lexington Kentucky USA; ^19^ Medical & Scientific Relations Alzheimer's Association Chicago Illinois USA; ^20^ Department of Neurology Washington University St Louis St Louis Missouri USA; ^21^ Dementia Research Centre, Institute of Neurology University College London London UK; ^22^ Department of Neurology Mayo Clinic Rochester Minnesota USA; ^23^ European Working Group of People With Dementia Alzheimer Europe Senningerberg Luxembourg; ^24^ Rush Alzheimer's Disease Center, Departments of Pathology and Neurological Sciences Rush University Medical School Chicago Illinois USA; ^25^ World Dementia Council London UK; ^26^ USF Health Byrd Alzheimer's Institute University of South Florida Tampa Florida USA; ^27^ European Working Group of People With Dementia (Supporter) Alzheimer Europe Senningerberg Luxembourg; ^28^ Neurochemistry Laboratory, Department of Laboratory Medicine, Neurodegeneration Amsterdam Neuroscience, Amsterdam UMC, Vrije Universiteit Amsterdam Amsterdam The Netherlands; ^29^ Alzheimer Center Amsterdam, Neurology Vrije Universiteit Amsterdam, Amsterdam UMC (VUmc) Amsterdam The Netherlands; ^30^ Neurodegeneration Amsterdam Neuroscience Amsterdam The Netherlands; ^31^ Epidemiology and Data Science Vrije Universiteit Amsterdam, Amsterdam UMC (VUmc) Amsterdam The Netherlands; ^32^ Department of Neurology and CNRMAJ Univ Rouen Normandie, Normandie Univ, Inserm U1245 and CHU Rouen Rouen France; ^33^ Section of Gerontology and Geriatric Medicine, Department of Internal Medicine Wake Forest University School of Medicine Winston‐Salem North Carolina USA; ^34^ Alzheimer's Disease Research, Center for Neurodegenerative Disorders, Department of Neurology IUSM/IUH Neuroscience Institute and Stark Neuroscience Research Institute, Indiana University School of Medicine Indianapolis Indiana USA

**Keywords:** Alzheimer's disease, clinical meaningfulness, dementia, health systems, nomenclature, patient‐caregiver dyad, real‐world data, shared decision‐making, therapies

## Abstract

Since 2016, the Alzheimer's Association and the Fondation Alzheimer have hosted Global Alzheimer's Leadership Series (GoALS) global think tanks, with world‐leading experts for innovative discussions to advance Alzheimer's disease (AD) research and care. The second GoALS think tank, held in June 2024 in Paris, focused on the relationship between biological changes and clinical manifestations of AD in the context of the evolving therapeutic landscape. Discussions spanned real‐world experiences of providers, patients, and their families, theoretical considerations, and health system challenges. The lived experience perspective was central to these discussions. The importance of shared decision‐making, clear and transparent communication, and the need for real‐world data to holistically support patients during their experiences were highlighted. This manuscript shares key insights from both the think tank meeting in Paris and a featured research session at the 2024 Alzheimer's Association International Conference that expanded the discussion themes for broader dissemination with the community.

## Highlights

- Advances in the field of Alzheimer's disease (AD) treatments led to the most recent GoALS global think tank, held in Paris in June 2024 and hosted by the Fondation Alzheimer and Alzheimer's Association, to focus on current understanding of the therapeutic dynamics between biological changes and the clinical manifestations of AD, including the evolving role of patients and caregivers in shared decision‐making about treatments, as well as the ongoing challenges of balancing risks and benefits in therapeutic interventions.
- Differing perspectives on the definition of Alzheimer's exist; however, they converge on the importance of the underlying biological substrate of AD and how it translates to clinical settings, including the importance of biomarkers, early treatment, and recognizing co‐pathologies and atypical clinical presentations. Further, there is agreement that at this point in time, clinical diagnosis of the disease is only appropriate and useful for medical decision‐making at the symptomatic stage.
- A critical component of managing treatment pathways is shared decision‐making and making these discussions meaningful to patients and caregivers.
- The evolving landscape of diagnosis and treatment tools in the AD and related dementias (ADRD) space demands continued collaboration among interested parties, including clinicians, researchers, policymakers, and patient advocacy groups. This will support the needs for continued development of clear communication, patient‐centered care, equitable access, and ongoing real‐world evidence generation to advance the larger ecosystem.

## 1 INTRODUCTION

The Global Alzheimer's Leadership Series (GoALS) is a joint initiative of the Fondation Alzheimer in France and the Alzheimer's Association in the United States to address critical gaps in the scientific and clinical understanding of Alzheimer's disease (AD) and related dementias (ADRD). Through a series of think tank events, GoALS brings together world‐leading experts to explore innovative research areas and develop opportunities to further these discussions with the research and clinical community, with the ultimate aim of advancing AD research and care. Launched in 2016, GoALS remains an ongoing partnership between the two organizations.

The AD field has made remarkable progress in expanding the therapeutic and diagnostic landscape. Notably, the emergence of anti‐amyloid beta (Aβ) therapies has transformed AD research and clinical care. Clinical trials of anti‐Aβ monoclonal antibodies have demonstrated significant reductions in brain Aβ deposits and a measurable slowing of the disease progression.^1^, ^2^, ^3^, ^4^The US Food and Drug Administration (FDA) has approved three anti‐Aβ therapies for AD,^2^, ^3^, ^4^, ^5^, ^6^, ^7^ with others under regulatory review. More recently, the European Medicines Agency's (EMA) Human Medicines Committee (CHMP) has recommended granting a marketing authorization on lecanemab for treating mild cognitive impairment (MCI) or mild dementia due to AD in patients who have only one or no copy of the apolipoprotein E (APOE) ε4 allele.^8^ In parallel, advancements in biomarker development, such as the advent of blood‐based biomarkers, have revolutionized the early diagnosis and monitoring of AD.^9^ Together, these diagnostic and therapeutic innovations underscore the importance of refining diagnosis and treatment strategies with real‐world evidence, which is critical to guiding clinical practice and improving patient outcomes.

The most recent GoALS global think tank, held in Paris in June 2024, focused on clarifying the current understanding of the dynamics between biological changes and the clinical manifestations of AD and on considerations of existing therapies, with emphasis on the lived experience. This includes the evolving role of patients and caregivers in treatment decisions, as well as the ongoing challenges of balancing risks and benefits in therapeutic interventions. Meeting participants, including an individual living with dementia and a family caregiver, shared experiences and perspectives throughout the entire discussion. Following that meeting, a featured research session at the 2024 Alzheimer's Association International Conference (AAIC) fostered a wider exchange of ideas.

In this paper, we summarize key insights from the 2024 GoALS meeting and the 2024 AAIC session, with an emphasis on patient and provider perspectives on the rapidly evolving treatment and diagnostic landscape of AD.

## 2 EVOLVING PERSPECTIVES ON AD DEFINITIONS AND NOMENCLATURE

Inconsistent nomenclature in defining AD has long posed challenges in communication among researchers, clinicians, patients, and the general public, affecting research efforts, clinical practice, and patients' understanding of the disease.^10^ The use of the term “Alzheimer's disease” in research and clinical practice is inconsistent, as it may refer to a clinical syndrome, a clinicopathologic complex, or a biological entity, conflating clinical and biological aspects of the disease. This inconsistency has significant implications for effective communication of critical concepts to patients, such as the clinical meaningfulness of AD therapies. Clear, consistent terminology is essential for ensuring that both clinicians and patients fully understand the nature of the disease and the implications of treatment.

In recent decades, the definition of AD has evolved from a historical clinical‐pathological construct to a biologically driven definition. Initially, AD diagnosis relied solely on the progression of clinical symptomatology, while a definitive diagnosis could only be confirmed through an autopsy. The 1984 National Institute of Neurological and Communicative Disorders and Stroke and the Alzheimer's Disease and Related Disorders Association (now known as the Alzheimer's Association) (NINCDS‐ADRDA) diagnostic criteria, which were based on observable clinical syndromes, have shaped clinical practice for decades.^11^ With advancements in biomarkers, however, the field has shifted to a biologically driven definition of AD. By 2011, a transformative shift had occurred in the field with the introduction of AD as a clinical biomarker construct by a workgroup of the National Institute on Aging and the Alzheimer's Association (NIA‐AA) and the International Work Group (IWG).^12^, ^13^, ^14^ At this time, the NIA‐AA workgroup, for example, published separate recommendations for diagnosing the clinical spectrum as preclinical AD, MCI due to AD, and dementia due to AD by incorporating biomarkers of brain Aβ deposition and downstream neurodegeneration to support the diagnosis of AD in symptomatic individuals.^15^, ^16^, ^17^ In 2018, another workgroup of the NIA‐AA proposed a further departure from the syndrome‐based approach and suggested employing the AT(N) construct (amyloid beta [A], tau [T], and neurodegeneration [N]) for diagnosing and staging AD based on biological markers only, detected through various imaging and cerebrospinal fluid (CSF) biomarkers.^18^, ^19^ This paradigm shift mirrors diagnostic approaches in other diseases, such as cancer, where biological evidence takes precedence over clinical symptoms. In Europe, the IWG in 2021 proposed criteria for AD diagnosis that included biomarkers but reserved the term “AD” for symptomatic stages and labeled preclinical disease as asymptomatic at risk for AD, reflecting the ongoing lack of international consensus on AD's definition.^20^

In 2024, further updates to the 2018 NIA‐AA research framework were proposed by another workgroup supported by the Alzheimer's Association.^21^ This update was driven by two major developments in the field: the introduction of novel FDA‐approved anti‐Aβ therapies that target the core disease pathology^1^, ^2^, ^3^ and the emergence of more accurate blood‐based biomarkers for AD.^9^ The revised criteria categorize biomarkers into three main categories: core biomarkers (Core 1 and Core 2 biomarkers, differentiated by the timing of abnormality onset and intended use), biomarkers of non‐specific processes involved in AD pathophysiology, and biomarkers of non‐AD co‐pathologies. Additionally, separate biological and clinical staging systems were proposed to reflect the disease's complexity. The revised criteria further recognize that AD does not exist in isolation; co‐pathologies and inflammatory processes often co‐occur, further complicating the diagnostic and therapeutic landscape. The revised criteria present objective criteria for diagnosing and staging AD based on its underlying biology, while acknowledging that they are subject to continued refinement.^21^

Shortly after the publication of the 2024 revised criteria by the Alzheimer's Association workgroup, the IWG responded with a revised framework similar to their prior work.^22^ The IWG argued for a clinical‐biological definition that requires the AD diagnosis only be made in symptomatic individuals with MCI or dementia. A diagnosis of presymptomatic AD could be made in individuals without symptoms but with the very uncommon genetic factors that impart close to 100% risk of developing symptoms. Furthermore, the IWG cautioned against labeling asymptomatic individuals as having AD, citing potential psychological harm. They introduced the term “at risk for AD” for asymptomatic individuals with positive AD biomarkers but low lifetime risk and “presymptomatic AD” for those with positive AD biomarkers and high lifetime risk.

Both the IWG and the Alzheimer's Association groups recognize the importance of the underlying biological substrate of AD and how it translates to clinical settings. They also highlight the importance of biomarkers, early treatment, and recognition of co‐pathologies and atypical clinical presentations. Furthermore, both sets of criteria agree that now, clinical diagnosis of the disease is only appropriate and useful for medical decision‐making at the symptomatic stage. However, the differences that pertain to the basic definition of AD in the presymptomatic or asymptomatic stage are critical for effective communication with patients and families and must be carefully clarified. Ultimately, a unified definition of AD is essential to advance the field and improve patient care.^23^ At the time of the think tank, the updated IWG had not been released; however, the discussion of the GoALS think tank was focused on a symptomatic population for consideration of treatment, clinical meaningfulness, and shared decision‐making.

In summary, the evolving definition and nomenclature of AD underscore the importance of clear communication, especially when discussing the benefits and risks of anti‐Aβ treatments with patients and their carers. The biological construct of AD is emerging but not yet widely understood by clinicians, most patients, carers, and the general public. The proposed terminologies from the Alzheimer's Association and IWG groups are generally aligned, with primary areas of divergence confined to the definition of preclinical and asymptomatic stages of the disease. This lack of full international consensus on the use of terms relating to the biological and clinical constructs of AD reinforces the need for collaborative initiatives to build consensus and for communication strategies that clarify the differences between the clinical symptomatology of AD and its underlying biology.

## 3 CLINICAL MEANINGFULNESS AND CONSIDERATIONS FOR AD THERAPIES

The introduction of anti‐Aβ therapies for AD has brought new complexities and challenges to clinical practice, particularly in effective communication of their benefits and risks to patients and their families. The information available so far is from carefully controlled clinical trials in a select group of patients – data are being gathered on real‐world use and are crucial to augmenting our understanding of the benefits and risks in different populations. Measuring the clinical meaningfulness of these therapies in early‐stage AD through short‐duration trials (typically 18 to 24 months) and clearly demonstrating it at the individual patient level is a challenging task.^24^ Therapy‐induced reduction of Aβ plaques in the brain, as shown by biomarkers, does not directly translate to proportional impacts in clinical outcomes on an individual level. This gap between biomarker changes and clinical outcomes challenges physicians in presenting and interpreting the clinical meaningfulness of AD therapies to patients and their caregivers.

To address some of these complexities, a workgroup of experts assembled by the Alzheimer's Association has examined what constitutes meaningful therapeutic benefits or slowing of disease progression while considering patients' and caregivers' perspectives.^24^ They highlight that measuring clinical meaningfulness goes beyond numbers, stressing the need to incorporate the expectations of patients and caregivers in defining what constitutes a clinically meaningful therapeutic outcome. For example, small improvements in cognitive score – such as a 0.5‐point improvement on the Clinical Dementia Rating Sum of Boxes (CDR‐SB) over 18 months – could translate into a substantial delay in cognitive and functional decline that is meaningful for patients and their caregivers at this stage of the disease. Furthermore, statistically significant changes observed over the course of an 18‐ to 24‐month trial may indicate meaningful long‐term benefits when extrapolated over subsequent years.^24^

Effective communication of statistical findings in clinical trials and their expected impact on patients and their families represents another challenge. For example, explaining the significance of a 30% slowing in the CDR‐SB score, as seen in anti‐Aβ therapy trials, can be difficult for those unfamiliar with the scale or statistics. To bridge this gap, the concept of “time saved” can be employed instead to better illustrate the slowing of disease progression.^25^ For instance, explaining that a 30% reduction in cognitive decline might translate into an additional 4 to 6 months of independence during an 18‐month treatment period can resonate more clearly with patients and their families.^26^ Developing clear communication materials and developing meaningful interactions with patients and caregivers could help in this respect. Moreover, mapping clinical meaningfulness to other more tangible functional measurements, such as instrumental and basic activities of daily living (IADL and ADL), can be helpful in demonstrating the effect of AD treatments on extending the time of independent functioning.^27^ Additionally, incorporating the development of prediction models, such as those aimed at forecasting cognitive decline on an individual level, may provide further insights.^28^

One key consideration in interpreting the clinical meaningfulness of AD therapies is that therapeutic outcomes may vary across disease stages and in the presence of mixed pathologies. While clinical trials are typically conducted on large groups of individuals, there is often a challenge in mapping group‐level effects to individual‐level outcomes.^24^ Patients may experience varying levels of benefit based on individual characteristics such as genetics, lifestyle, co‐pathology, psychological and environmental factors, and resiliency factors. Therefore, it is essential to manage expectations around the individual clinical impact of AD therapies depending on individual patient characteristics.^24^ Our understanding of these drugs so far is based on information from the initial trials; this will expand as real‐world data accrue.^29^ For now, it is crucial that we be transparent with each individual – where there is a knowledge gap, state that clearly. The process of learning how to discuss these therapies will evolve as our knowledge increases. Ultimately, the concept of clinical meaningfulness is relevant to various interested parties – such as patients, care partners, clinicians, clinical trialists, regulators, and payers – while the definition may vary across interest holders.^30^ However, at the individual level, a meaningful benefit is also related to potential harm. Communication of anti‐Aβ therapy must include an explanation of the likelihood of actual harm, primarily in connection with amyloid‐related imaging abnormalities (ARIA). Like the challenges of explaining a statistical benefit for clinical symptoms, effectively communicating information about an adverse effect that is primarily without symptoms, but rarely with severe symptoms, poses an additional challenge for clinicians and patients.

The risks associated with anti‐Aβ therapies must be communicated clearly to patients and their families. Anti‐Aβ therapies carry a unique adverse event profile, known as ARIA, which requires ongoing surveillance measures, including regular magnetic resonance imaging (MRI) scans.^31^ Although often asymptomatic, these imaging changes are characterized by edema and/or hemorrhage and in some persons can lead to significant symptoms, disability, and even death. Certain patients, particularly APOE ε4 homozygous individuals, are at increased risk for ARIA, spurring ongoing debates in the field about the treatment of these individuals.^32^ Other ARIA risk factors include MRI evidence of cerebral amyloid angiopathy (CAA), treatment duration and dosing, higher amyloid positron emission tomography (PET) burden, elevated baseline blood pressure, white matter hyperintensity (WMH) severity, vascular comorbidities, and anticoagulant use.^32^, ^33^

These considerations underscore the need for transparent conversations about genetic testing, eligibility, and safety that inform treatment decisions, as well as the ethical and psychological implications of disclosing APOE homozygosity information to patients and their families. An APOE homozygosity status may impact eligibility for treatment with anti‐Aβ therapies while also raising concerns about the speed of the progression of the disease, as well as family implications. Therefore, such discussions with patients and their families should be approached with transparency, addressing the risks and benefits of both genetic testing and treatment options. It is also important to acknowledge the uncertainties in our knowledge and that anti‐Aβ therapies should not be viewed as an “all‐or‐nothing” approach. Alternatives, such as non‐pharmacological interventions and supportive therapies, should also be considered as part of a holistic treatment approach to address patients’ broader needs. Ultimately, ensuring that patients and caregivers are well informed about both risks and benefits in the context of their own personal medical and genetic histories is key to supporting informed decision‐making.

In summary, the individual perspective on the clinical benefits of AD therapies remains critical. While clinical meaningfulness is often demonstrated statistically through standardized scales, a deeper understanding of what this means to patients, including the influence of cultural differences, is necessary. To address this, more inclusive recruitment strategies are required, alongside enhanced measurements of patient‐reported outcomes (PROs)^34^ and participant/dyad experiences in clinical trials. Advocacy groups, like the Alzheimer's Association and Alzheimer Europe, are already working with patient groups for better integration of lived experiences of AD into clinical trials and clinical assessment methods. Public involvement can also add valuable insights from the lived experience of AD, supporting the design of more inclusive clinical trials and the development of endpoints that are meaningful for patients and carers. Moreover, critical gaps remain in our understanding of the long‐term impact of anti‐Aβ therapies on disease progression. Continued post‐marketing surveillance and robust longitudinal studies will be essential to further clarify the benefits and risks of extended therapy duration in broader populations.

## 4 CONSIDERATIONS FOR CLINICAL FLOW AND SHARED DECISION‐MAKING IN AD THERAPIES

The advent of anti‐Aβ therapies has underscored the need for rethinking the clinical flow and patient navigation through treatment pathways. In this context, one of the central themes that emerged from the GoALS discussions was the importance of information, education, and communication. It was noted that there is a need for accurate and accessible information for both patients and healthcare professionals. Although substantial data on the efficacy and safety of these therapies exist, there remain significant gaps, particularly in the real‐world context, such as managing patients with different backgrounds, co‐existing conditions or addressing differences related to sex or age. The challenge is not only in gathering this information but also in ensuring it is presented clearly and accurately, while acknowledging the knowledge gaps and the evolving nature of treatment approaches to enable informed, shared decision‐making. Additionally, enhanced educational material and resources for both healthcare professionals and the public are essential as these new therapies transform AD treatment.^35^

### 4.1 Shared decision‐making: patient and caregiver perspective

A critical component of managing treatment pathways is shared decision‐making and making these discussions meaningful to patients and caregivers. Shared decision‐making is a collaborative process where a person receiving care and a healthcare professional work together to make decisions about treatment and care.^36^ Such discussions require understanding and respecting patients’ and caregivers' preferences, goals, and lifestyles. No two patients are alike, and each brings a unique set of values and priorities to their care. While clinical trial data provide a statistical basis for understanding treatment outcomes, discussions with patients, their families, and caregivers need to be personalized. For example, some patients may prioritize retaining their ability to work or engage in independent hobbies like cooking, while others may value additional months of quality life with family members and friends. It is equally important that information about benefits and risks be presented transparently and accessibly, without overwhelming the patients' and caregivers' ability to balance their own hopes and fears. Ensuring that these discussions are patient‐centered and that individuals receive the right information, tools, and support is essential to guiding them through the decision‐making process.

Furthermore, navigating discussions to support shared decision‐making requires an understanding of the caregiver's perspective. Caregivers, particularly those in close dyadic relationships with patients, often provide unique insights into quality of life and disease progression that complement patients’ perspectives. Creating safe environments that allow caregivers to share their experiences in a supportive environment is critical, as is creating safe spaces for both patients and caregivers to engage in meaningful conversations. Ultimately, providing such personalized information and discussions to patients and caregivers requires a substantial amount of time, and healthcare systems need to allocate sufficient resources to support such in‐depth discussions.

It is also important to note that attitudes toward monoclonal anti‐Aβ therapies vary among patients, caregivers, and professionals. A 2021 survey in the United Kingdom (UK) found that while 70% of old‐age psychiatrists [this refers to the UK‐named specialty care] did not support early diagnosis for dementia, 70% of patients favored it, indicating a disconnect in perspectives.^37^ While these attitudes are now evolving as science advances, there remains a need for education to build an understanding of the benefits and considerations (i.e., discrimination, insurance, and other aspects) of early, accurate, and biologically confirmed diagnosis. Additionally, professionals may feel under‐resourced and lacking the necessary skills to implement advanced diagnostic tools and emerging treatments or to address the downstream social consequences of diagnosis, underscoring the need for training and resource development. Unpublished data presented at 2024 AAIC from interviews with patients, caregivers, and doctors in Europe also showed significant variability in what patients and caregivers want to know about their options. Similarly, professionals’ attitudes toward the risks and benefits of anti‐Aβ therapies showed significant variability. Together, these findings highlight the importance of education and communication about these varied perspectives to support shared decision‐making.

Engaging in meaningful discussions with patients and caregivers to support shared decision‐making also emphasizes the importance of recognizing the evolving landscape of AD treatment. Healthcare professionals need to be transparent about the limitations of current knowledge, acknowledging both the rapid advancements and uncertainties in the field. The need to train and educate providers was noted, although the specifics for how this should and could be carried out are specific for the environment in which the provider is engaged in care. There are likely significant differences in needs between the United States and Europe and other parts of the world. These differences may reflect variations in healthcare system structures, access to care, reimbursement models, and timelines for the adoption of new therapies. For example, funding limitations experienced by national healthcare systems in European countries may preclude the widespread use of more expensive medications. Additionally, we need to realize that treatment decisions for AD are not always binary (treatment or no treatment): As highlighted by an individual with AD at the GoALS meeting, some individuals may prefer to pursue other therapeutic avenues or may wish to discontinue treatment, reinforcing the need for individualized approaches to decision‐making, treatment, and care.

### 4.2 Navigating the clinical flow and access issues

In countries where anti‐Aβ therapies are being marketed, access to treatment and advanced biomarkers remains challenging, with disparities in the availability of infusion centers and trained specialists across centers. In the United States, many neurologists, psychiatrists, and geriatricians face barriers due to limited resources and staff, and smaller clinics or rural centers may lack the resources to provide these treatments efficiently. In contrast, larger private centers equipped with onsite infusion facilities offer more seamless access. Academic centers also face variability in access, with some institutions adopting comprehensive, multidisciplinary approaches with weekly consensus conferences to determine eligibility, while others lack specialist neurology practices, and providers make individual prescribing decisions alone. While such multidisciplinary approaches have shown promise in optimizing patient care in practice, it is critical to find ways to implement such collaborative models broadly, particularly in resource‐limited settings such as primary care.

Some academic centers in the United States have introduced “brain health navigators” – specialized professionals who guide patients through the diagnostic and treatment process, simplifying the journey and providing consistent support from diagnosis through eligibility assessment and treatment monitoring. However, such resources are not universally available, resulting in considerable disparities in how and when patients can receive therapies. Furthermore, access to advanced diagnostic tools such as CSF analysis or PET scans is limited, reflecting a broad resource challenge. Post‐diagnosis support also remains insufficient for many patients, leaving individuals and their caregivers without adequate resources to navigate their condition. Addressing these issues will require developing more equitable healthcare systems that ensure access to diagnostic tools, therapies, and post‐diagnostic care for all patients. Monitoring individuals on anti‐Aβ therapies requires careful management, with frequent MRI brain scans and follow‐up by trained nurses or navigators, which will require additional resources.

An example from Japan shared at the GoALS meeting illustrated how structured policies and reimbursement support can help bridge some of these gaps. Lecanemab was launched in Japan in 2023, with over a thousand patients receiving treatment across 328 clinics by June 2024. To enhance safety, prescribing is restricted to select experts and centers, with mandatory post‐marketing surveillance for all prescribed cases. In addition, an academic “AD registry study” takes responsibility for APOE genotyping performed in a clinical research setting prior to individual eligibility approval. Coverage from Japan's national health insurance includes lecanemab, amyloid PET, or CSF biomarker tests that support access. Furthermore, the Japanese high‐cost medical expense benefits waive costs >1750 USD per year for elderly patients, which reduces patient expenses. However, APOE genotyping, a critical part of treatment planning, is not yet covered clinically and may take 2 years to gain approval. This example highlights the impact of a strong regulatory framework and reimbursement policies in facilitating access, though gaps remain.

It is also important to recognize that treatment for AD is multifaceted, extending beyond anti‐Aβ therapies. Stronger collaboration between primary, secondary, and tertiary care is needed to ensure that appropriate experts are present to discuss all treatment options. A wide range of therapeutic approaches should be considered, from symptomatic therapies to psychosocial support and multidomain interventions. Patients who are not eligible for anti‐Aβ treatments or those who forgo the decision to use them should not be left without adequate care options. Avoiding a two‐tiered system, in which one group receives advanced therapies and the other does not, will require intentional efforts to ensure equitable access to evaluation, diagnosis, counseling, and ongoing management across care settings. Care pathways should ensure that all patients receive comprehensive and equitable treatment, regardless of their eligibility for specific therapies.

Finally, knowledge gaps regarding comorbidities and variations in treatment response across different populations highlight the importance of expanding research to include low‐ and middle‐income countries (LMICs) and under‐represented ethnoracialized groups. In LMICs, challenges include the lack of infrastructure to implement biomarker‐based diagnostics, which limits the applicability of the biological definition of AD. The potential use of blood‐based biomarkers in the future could help address this issue. Further research is also needed to assess the reliability and effectiveness of various biomarkers in LMIC populations. These efforts will help ensure that future treatment guidelines are inclusive and applicable to a broader range of patients. Globally, the collection and sharing of real‐world data through registries is crucial. Learning from ongoing experiences in real‐world settings and drawing from knowledge in related fields will help refine therapeutic approaches and support the development of best practices.

## 5 UNKNOWNS AND GAPS IN DEFINING TREATMENT INITIATION AND DISCONTINUATION

Anti‐Aβ therapies represent a critical milestone in the AD field, paving the way toward a future focused on personalized medicine. As we stand at the forefront of these advancements, the field is presented with numerous research opportunities to address key unknowns and knowledge gaps that could shape the future of AD treatment and diagnosis.

One important knowledge gap is determining who benefits most from these therapies and establishing clear criteria for treatment initiation. Findings from the TRAILBLAZER trial, for instance, have shown that patients with low to medium tau levels, as measured by tau‐PET imaging, experience greater clinical benefits than those with high tau burden.^4^ This highlights the potential of tau‐PET in selecting patients most likely to respond to donanemab therapy. Tau‐PET is not widely accessible and is not fully validated for routine clinical use, limiting its applicability in real‐world settings. Consequently, there is a need to identify scalable, alternative biomarkers to determine the optimal disease stage for treatment initiation.

Beyond the disease stage, the complexity and heterogeneity of AD pose another challenge to identifying individuals most likely to benefit from treatments.^38^ Recent work has identified five molecular subtypes of AD based on CSF proteomic analysis. These subtypes – characterized by neuronal hyperplasticity, innate immune activation, RNA dysregulation, choroid plexus dysfunction, and blood–brain barrier dysfunction – may help refine patient stratification for treatment.^39^ In addition, it is important to note that the majority of individuals with AD have mixed co‐existing pathologies^40^, ^41^, ^42^ with additional contributing factors such as Limbic‐predominant age‐related TDP‐43 encephalopathy (LATE), cerebrovascular disease, or Lewy body pathology. Currently, there are no established biomarkers for many of these co‐existing pathologies, and it remains unclear how current treatments perform in the context of specific mixed pathologies. Overall, the complexity of AD is driven by the convergence of multiple factors, such as genetic, environmental, and pathogenic pathways.^43^ These insights highlight the need for multifaceted approaches to better understand the heterogeneity of AD and how it impacts treatment effectiveness or the development of adverse effects, which could enhance personalized treatment. Given the relatively homogeneous populations selected for the trials that led to the approval of anti‐Aβ therapies, it remains unknown what the clinical benefit and risk profile will be in “real‐world” populations. This will require close monitoring of outcomes from registries^44^ and the experience of clinics enrolling large numbers of participants.^29^ However, these sources may remain subject to bias and may not fully capture the treatment benefits and risks across the AD heterogeneity landscape.

In addition to determining who should receive treatment, the field must also address the optimal criteria for discontinuing therapy. Unlike symptomatic therapies like cholinesterase inhibitors, for which biomarkers of target engagement are lacking, disease‐modifying treatments provide the opportunity to objectively assess treatment effects through biomarker measurements.^35^ Drawing lessons from oncology, the AD field can incorporate biomarker data to guide decisions on when to stop treatment. For instance, after a defined treatment period, clinicians could evaluate whether amyloid has been sufficiently cleared. If it has, patients could transition to a new disease entity with a proposed name of treatment‐related amyloid clearance (TRAC). TRAC refers to individuals who have lower levels of amyloid following treatment but still have AD. If amyloid levels have not been reduced sufficiently, continuation of therapy may not be justified. However, at this moment, this approach is limited by the varying sensitivity of different biomarkers and may require longitudinal studies to assess the true long‐term biological and clinical effects of these therapies. Importantly, discontinuing therapy should not merely be viewed as stopping a chemical intervention but rather as a carefully considered decision within a broader treatment framework while considering patient and caregiver perspectives.

The unresolved questions of whether and when to stop treatment, implement maintenance therapy, perform post‐clearance (TRAC) monitoring, and how or when to restart therapy necessitate further clinical utility studies. Further work is needed to understand what predicts response or non‐response. Real‐world evidence is crucial for understanding the long‐term trajectory of AD under treatment, assessing side effects, and identifying which patients derive the greatest benefit. Moreover, clinical utility studies will aid in the development of alternative biomarkers for optimal treatment timing, the identification of molecular subtypes with the highest likelihood of treatment success and lowest risks, and the establishment of stopping criteria. Ultimately, biomarkers offer the potential for more precise and objective decision‐making in both initiating and discontinuing treatment with disease‐modifying therapies. However, as with any groundbreaking innovation, there is still much to learn.

## 6 SUMMARY

Since its inaugural meeting in 2016, the GoALS model of discussion has served as a unique vehicle to advance strategic priorities in AD research and care. Over this period, the AD field has achieved remarkable progress, with anti‐Aβ therapies transforming treatment approaches and blood‐based biomarkers revolutionizing AD diagnostics. Through the most recent GoALS meeting in 2024, discussion focused on these breakthroughs emphasize the need to refine diagnostic and treatment paradigms, informed by real‐world evidence, to guide clinical decision‐making and improve patient outcomes.

The meeting discussants underscored the critical need for clear, harmonized nomenclature and definitions in the AD space to meet the expectations of patients and their families. Communication barriers may hinder the effective implementation of therapies, delay diagnosis, and create confusion among patients, caregivers, and healthcare providers, as well as payers and other key players in this ecosystem.

A key theme of the discussions centered on the clinical meaningfulness of AD therapies and the importance of shared decision‐making frameworks. Translating clinical trial results into outcomes that are tangible for patients is complex, emphasizing that the individual perspective on the clinical benefits of AD therapies is critical. In this context, there remains a need to develop more inclusive recruitment strategies alongside enhanced measurements of patient‐reported outcomes (PROs) in clinical trials. Additionally, the implementation of shared decision‐making frameworks emerged as a key priority that would enable patients and caregivers to make informed choices based on personal goals, risk tolerance, and lifestyles. Transparent information, education, and communication about both advancements and knowledge gaps are key, supported by personalized discussions that respect individual values and preferences.

There are systemic and logistical barriers to the successful implementation of AD therapies in clinical practice such as resource limitations, although learning from one another is a powerful approach to helping address these barriers. Persistent disparities in access to early‐stage screening, advanced diagnostics, infusion centers, and specialized care continue to pose challenges, exacerbating inequities in treatment delivery. Addressing these issues will require the development of more equitable healthcare systems that ensure access to diagnostic tools, therapies, and post‐diagnostic care for all patients. Importantly, discussants emphasized the critical need for ongoing research and real‐world data to address knowledge gaps, including optimal initiation and discontinuation criteria for AD therapies, the role of mixed pathologies, and the long‐term impacts of treatment.

Opportunities for further dialogue among different interested interdisciplinary parties and ensuring the lived‐experience perspective is accounted for were highlighted, as were opportunities to further inform our understanding of the biological continuum. Specific action items include the following:

- Fostering a discussion at the 2024 AAIC that included the lived‐experience perspective and voice;
- Sharing the outcome of the discussion through a manuscript and accessible related communications, such as blogs with the communities of the Fondation Alzheimer and the Alzheimer's Association;
- Engaging in ongoing discussion and discourse about the biological definition of AD (Alzheimer's Association clinical diagnosis and staging criteria) and others, such as the IWG at the 2025 AAIC ;
- Continuing to facilitate opportunities for sharing and learning among international clinicians and providers, such as through the Alzheimer's Association Network for Treatment and Diagnosis (ALZ‐NET) and its international arm, ALZ‐NET International; and
- Encouraging engagement with lived experience perspective in these discussions and identifying and developing resources to support clinicians in discussion with patients throughout the entire care journey (in line with emerging science and growing understanding).

In conclusion, the evolving landscape of AD diagnosis and treatment demands continued collaboration among interested parties, including clinicians, researchers, policymakers, and patient advocacy groups, and clear communication, patient‐centered care, equitable access, and ongoing real‐world evidence generation will be essential.

## CONFLICT OF INTEREST STATEMENT

At the time of this meeting and manuscript preparation/ submission, the authors declared the following conflicts: MCC: Full‐time employee of the Alzheimer's Association; Child is a graduate student in neurosciences at the University of Southern California. HS: Full‐time employee of the Alzheimer's Association; spouse works at Abbott Labs in an unrelated field. SM: Full‐time employee of the Alzheimer's Association. CJM: Funded by the UCLH Biomedical Research Centre; is on the advisory board/consults for Biogen, Roche, WAVE, IONIS, Prevail, Lilly, and Eisai; has been awarded a grant from Biogen for the development of ultrafast MRI methodology; and has participated in sponsored symposia by Biogen, Roche, EISAI, IONIS, and Lilly. ACB: Full‐time employee of Alzheimer Europe (AE) and represents AE in the Patients’ and Consumers’ Working Party of the European Medicines Agency. AGS: Receives research support from Biogen, Eisai, Eli Lilly, Vivoryon, Cassava, Janssen, Bristol‐Myers Squibb, Alzheimer's Association, and Alzheimer's Clinical Trial Consortium. DW: No conflict of interest to disclose. LTG: Receives research funds from the National Institutes of Health (NIH) and Rainwater Charitable Foundation. Has been awarded a grant from Roche, via Weill Neurohub, to investigate synapse changes in neurodegenerative disease; is a member of the Alzheimer's Association Medical & Scientific Advisory Group (MSAG). Received honoraria for educational material from Medscape Inc. and Otkusa; received consulting honorarium from Guidepoint Inc. GAJ: Funded by the NIH R01AG075959, R01AG061111, U19NS120384, P01AG078116, P30AG072946, R01NS116058, U54TR001998, U24AG057437; conducts contract research for AbbVie, Cassava, Cognition, Eisai, Eli Lilly, Novo Nordisk, and Vivoryon. CRJ: Employed by the Mayo Clinic; receives grant funding from NIH (R37 AG011378, R01 AG041851), the Alexander family professorship, and the GHR Foundation; within the past 36 months, served on a Data Safety & Monitoring Board (DSMB) for Roche pro bono; no payments to the individual or institution were involved; has received funding from the Alzheimer's Association for travel to scientific meetings. MD: No conflict of interest to disclose. SR and BT: No conflict of interest to disclose. RCP: Funded by NIH, consults for Roche, Genentech, Eli Lilly, Eisai, Novo Nordisk, and Novartis; receives royalties from Oxford University Press and UpToDate. WF: Research programs of Wiesje van der Flier have been funded by ZonMW, NWO, EU‐JPND, EU‐IHI, Alzheimer Nederland, Hersenstichting CardioVascular Onderzoek Nederland, Health∼Holland, Topsector Life Sciences & Health, stichting Dioraphte, Gieskes‐Strijbis fonds, stichting Equilibrio, Edwin Bouw fonds, Pasman stichting, stichting Alzheimer & Neuropsychiatrie Foundation, Philips, Biogen MA Inc, Novartis‐NL, Life‐MI, AVID, Roche BV, Eli‐Lilly‐NL, Fujifilm, Eisai, Combinostics. WF holds the Pasman Chair. WF is recipient of ABOARD, which is a public‐private partnership receiving funding from ZonMW (73305095007) and Health∼Holland, Topsector Life Sciences & Health (PPP‐allowance; LSHM20106). WF is recipient of TAP‐dementia (www.tap‐dementia.nl), receiving funding from ZonMw (10510032120003). TAP‐dementia receives co‐financing from Avid Radiopharmaceuticals and Amprion. All funding is paid to her institution. WF has been an invited speaker at Biogen MA Inc., Danone, Eisai, WebMD Neurology (Medscape), NovoNordisk, Springer Healthcare, and European Brain Council. All funding is paid to her institution. WF is consultant to Oxford Health Policy Forum CIC, Roche, Biogen MA Inc., and Eisai. All funding is paid to her institution. WF served on advisory boards of Biogen MA Inc., Roche, and Eli Lilly. WF is a member of the steering committee of EVOKE/EVOKE+ (NovoNordisk). All funding is paid to her institution. WF is a member of the steering committee of Project Alzheimer’s Value Europe (PAVE) and Think Brain Health. WF was associate editor of *Alzheimer's Research & Therapy* in 2020/2021. WF is associate editor at Brain. CT: CET has research contracts with Acumen, ADx Neurosciences, AC‐Immune, Alamar, Aribio, Axon Neurosciences, Beckman‐Coulter, BioConnect, Bioorchestra, Brainstorm Therapeutics, Celgene, Cognition Therapeutics, EIP Pharma, Eisai, Eli Lilly, Fujirebio, Instant Nano Biosensors, Novo Nordisk, Olink, PeopleBio, Quanterix, Roche, Toyama, and Vivoryon. She is editor in chief of *Alzheimer's Research and Therapy* and serves on the editorial boards of *Molecular Neurodegeneration*, *Neurology: Neuroimmunology & Neuroinflammation*, and *Medidact Neurologie*/Springer and serves on a committee to define guidelines for cognitive disturbances and on one for acute neurology in the Netherlands. She has had consultancy/speaker contracts for Aribio, Biogen, Beckman‐Coulter, Cognition Therapeutics, Eli Lilly, Merck, Novo Nordisk, Olink, Roche, and Veravas. JAS: Funding from NIH, consultant for Meilleur Technologies (Enigma), Eli Lilly, Alnylam, Lantheus Cerveau. OH: Currently an employee of Eli Lilly (at the time of this submission, but not at the time of the workshop and initial manuscript preparation) and Lund University; he previously acquired research support (for Lund University) from AVID Radiopharmaceuticals, Biogen, C2N Diagnostics, Eli Lilly, Eisai, Fujirebio, GE Healthcare, and Roche. In the past 2 years, he has received consultancy/speaker fees from Alzpath, BioArctic, Biogen, Bristol Myers Squibb, Eisai, Eli Lilly, Fujirebio, Merck, Novartis, Novo Nordisk, Roche, Sanofi, and Siemens. Author disclosures are available in the Supporting Information.

## Supporting information




**Supporting Information**: alz71536‐sup‐0001‐ICMJE.pdf

## References

[1] Mintun MA , Lo AC , Duggan Evans C , et al. Donanemab in Early Alzheimer's Disease. N Engl J Med. 2021;384(18):1691‐1704. doi:10.1056/NEJMoa2100708 33720637

[2] van Dyck CH , Swanson CJ , Aisen P , et al. Lecanemab in Early Alzheimer's Disease. N Engl J Med. 2023;388(1):9‐21. doi:10.1056/NEJMoa2212948 36449413

[3] Budd Haeberlein S , Aisen PS , Barkhof F , et al. Two Randomized Phase 3 Studies of Aducanumab in Early Alzheimer's Disease. J Prev Alzheimers Dis. 2022;9:197‐210. doi:10.14283/jpad.2022.30 35542991

[4] Sims JR , Zimmer JA , Evans CD , Lu M , Ardayfio P , Sparks J , et al. Donanemab in Early Symptomatic Alzheimer Disease: The TRAILBLAZER‐ALZ 2 Randomized Clinical Trial. JAMA. 2023;330(6):512‐527. doi:10.1001/jama.2023.13239 37459141 PMC10352931

[5] Commissioner O of the . FDA Converts Novel Alzheimer's Disease Treatment to Traditional Approval. FDA. 2023. https://www.fda.gov/news‐events/press‐announcements/fda‐converts‐novel‐alzheimers‐disease‐treatment‐traditional‐approval. accessed July 10, 2023.

[6] Research C for DE and. FDA approves treatment for adults with Alzheimer's disease. FDA 2024.

[7] Commissioner O of the. FDA Grants Accelerated Approval for Alzheimer's Drug. FDA 2021. https://www.fda.gov/news‐events/press‐announcements/fda‐grants‐accelerated‐approval‐alzheimers‐drug (accessed September 24, 2024)

[8] Leqembi recommended for treatment of early Alzheimer's disease | European Medicines Agency (EMA) 2024. https://www.ema.europa.eu/en/news/leqembi‐recommended‐treatment‐early‐alzheimers‐disease (accessed December 10, 2024)

[9] Hansson O , Edelmayer RM , Boxer AL , et al. The Alzheimer's Association appropriate use recommendations for blood biomarkers in Alzheimer's disease. Alzheimers Dement. 2022;18(12):2669‐2686. doi:10.1002/alz.12756 35908251 PMC10087669

[10] Petersen RC , Weintraub S , Sabbagh M , et al. A New Framework for Dementia Nomenclature. JAMA Neurol. 2023;80(12):1364‐1370. doi:10.1001/jamaneurol.2023.3664 37843871 PMC12136284

[11] McKhann G , Drachman D , Folstein M , Katzman R , Price D , Stadlan EM . Clinical diagnosis of Alzheimer's disease: report of the NINCDS‐ADRDA Work Group under the auspices of Department of Health and Human Services Task Force on Alzheimer's Disease. Neurology. 1984;34(7):939‐944. doi:10.1212/wnl.34.7.939 6610841

[12] Jack CR , Albert MS , Knopman DS , et al. Introduction to the recommendations from the National Institute on Aging‐Alzheimer's Association workgroups on diagnostic guidelines for Alzheimer's disease. Alzheimers Dement. 2011;7(3):257‐262. doi:10.1016/j.jalz.2011.03.004 21514247 PMC3096735

[13] Dubois B , Feldman HH , Jacova C , Dekosky ST , Barberger‐Gateau P , Cummings J , et al. Research criteria for the diagnosis of Alzheimer's disease: revising the NINCDS‐ADRDA criteria. Lancet Neurol. 2007;6(8):734‐746. doi:10.1016/S1474-4422(07)70178-3 17616482

[14] Dubois B , Feldman HH , Jacova C , et al. Revising the definition of Alzheimer's disease: a new lexicon. Lancet Neurol. 2010;9(11):1118‐1127. doi:10.1016/S1474-4422(10)70223-4 20934914

[15] Albert MS , DeKosky ST , Dickson D , et al. The diagnosis of mild cognitive impairment due to Alzheimer's disease: recommendations from the National Institute on Aging‐Alzheimer's Association workgroups on diagnostic guidelines for Alzheimer's disease. Alzheimers Dement. 2011;7(3):270‐279. doi:10.1016/j.jalz.2011.03.008 21514249 PMC3312027

[16] McKhann GM , Knopman DS , Chertkow H , et al. The diagnosis of dementia due to Alzheimer's disease: recommendations from the National Institute on Aging‐Alzheimer's Association workgroups on diagnostic guidelines for Alzheimer's disease. Alzheimers Dement. 2011;7(3):263‐269. doi:10.1016/j.jalz.2011.03.005 21514250 PMC3312024

[17] Sperling RA , Aisen PS , Beckett LA , et al. Toward defining the preclinical stages of Alzheimer's disease: recommendations from the National Institute on Aging‐Alzheimer's Association workgroups on diagnostic guidelines for Alzheimer's disease. Alzheimers Dement. 2011;7(3):280‐292. doi:10.1016/j.jalz.2011.03.003 21514248 PMC3220946

[18] Jack CR , Bennett DA , Blennow K , et al. A/T/N: an unbiased descriptive classification scheme for Alzheimer disease biomarkers. Neurology. 2016;87(5):539‐547. doi:10.1212/WNL.0000000000002923 27371494 PMC4970664

[19] Jack CR , Bennett DA , Blennow K , et al. NIA‐AA Research Framework: toward a biological definition of Alzheimer's disease. Alzheimers Dement 2018;14(4):535‐562. doi:10.1016/j.jalz.2018.02.018 29653606 PMC5958625

[20] Dubois B , Villain N , Frisoni GB , et al. Clinical diagnosis of Alzheimer's disease: recommendations of the International Working Group. Lancet Neurol. 2021;20(6):484‐496. doi:10.1016/S1474-4422(21)00066-1 33933186 PMC8339877

[21] Jack CR , Andrews JS , Beach TG , et al. Revised criteria for diagnosis and staging of Alzheimer's disease: alzheimer's Association Workgroup. Alzheimers Dement. 2024; 20(8):5143‐5169. doi:10.1002/alz.13859 38934362 PMC11350039

[22] Dubois B , Villain N , Schneider L , et al. Alzheimer Disease as a Clinical‐Biological Construct—An International Working Group Recommendation. JAMA Neurol. 2024;81(12):1304‐1311. doi:10.1001/jamaneurol.2024.3770 39483064 PMC12010406

[23] Petersen RC , Mormino E , Schneider JA . Alzheimer Disease‐What's in a Name?. JAMA Neurol. 2024;81(12):1245‐1246. doi:10.1001/jamaneurol.2024.3766 39483063

[24] Petersen RC , Aisen PS , Andrews JS , et al. Expectations and clinical meaningfulness of randomized controlled trials. Alzheimers Dement. 2023;19(6):2730‐2736. doi:10.1002/alz.12959 36748826 PMC11156248

[25] Dickson SP , Wessels AM , Dowsett SA , et al. Time Saved’ As a Demonstration of Clinical Meaningfulness and Illustrated Using the Donanemab TRAILBLAZER‐ALZ Study Findings. J Prev Alzheimers Dis. 2023;10(3):595‐599. doi:10.14283/jpad.2023.50 37357301

[26] Wang G , Cutter G , Oxtoby NP , et al. Statistical considerations when estimating time‐saving treatment effects in Alzheimer's disease clinical trials. Alzheimers Dement. 2024;20(8):5421‐5433. doi:10.1002/alz.14035 39030751 PMC11350030

[27] Hartz SM , Schindler SE , Streitz ML , et al. Assessing the clinical meaningfulness of slowing CDR‐SB progression with disease‐modifying therapies for Alzheimer disease. medRxiv. Preprint. 2024. doi:10.1101/2024.07.16.24310511 PMC11822626 39949872

[28] Van Der Veere PJ , Hoogland J , Visser LNC , et al. Predicting cognitive decline in amyloid‐positive patients with mild cognitive impairment or mild dementia. Neurology. 2024;103(3):e209605. doi:10.1212/WNL.0000000000209605 38986053 PMC11238942

[29] Paczynski M , Hofmann A , Posey Z , et al. lecanemab treatment in a specialty memory clinic. JAMA Neurol. 2025;82(7):655‐665. doi:10.1001/jamaneurol.2025.1232 40354064 PMC12070285

[30] Tochel C , Smith M , Baldwin H , et al. What outcomes are important to patients with mild cognitive impairment or Alzheimer's disease, their caregivers, and health‐care professionals? A systematic review. Alzheimers Dement Amst Neth. 2019;11:231‐247. doi:10.1016/j.dadm.2018.12.003 PMC6411507 30906845

[31] Sperling RA , Jack CR , Black SE , et al. Amyloid‐related imaging abnormalities in amyloid‐modifying therapeutic trials: recommendations from the Alzheimer's association research roundtable workgroup. Alzheimers Dement. 2011;7(4):367‐385. doi:10.1016/j.jalz.2011.05.2351 21784348 PMC3693547

[32] Hampel H , Elhage A , Cho M , Apostolova LG , Nicoll JAR , Atri A . Amyloid‐related imaging abnormalities (ARIA): radiological, biological and clinical characteristics. Brain J Neurol. 2023;146(11):4414‐4424. doi:10.1093/brain/awad188 PMC10629981 37280110

[33] Greenberg SM , Bax F , van Veluw SJ . Amyloid‐related imaging abnormalities: manifestations, metrics and mechanisms. Nat Rev Neurol. 2025;21(4):193‐203. doi:10.1038/s41582-024-01053-8 39794509

[34] Mank A , Van Maurik IS , Bakker ED , Van De Glind EMM , Jönsson L , Kramberger MG , et al. Identifying relevant outcomes in the progression of Alzheimer's disease; what do patients and care partners want to know about prognosis?. Alzheimers Dement (N Y). 2021;7(1):e12189. doi:10.1002/trc2.12189 34458555 PMC8377775

[35] Cummings J . Anti‐amyloid monoclonal antibodies are transformative treatments that redefine Alzheimer's disease therapeutics. Drugs. 2023;83(7):569‐576. doi:10.1007/s40265-023-01858-9 37060386 PMC10195708

[36] Montori VM , Ruissen MM , Hargraves IG , Brito JP , Kunneman M . Shared decision‐making as a method of care. BMJ Evid‐Based Med. 2023;28(4):213‐217. doi:10.1136/bmjebm-2022-112068 PMC10423463 36460328

[37] Are we ready to deliver disease modifying treatments? Old Age Psychiatrists’ views on diagnosing and treating Alzheimer's disease before dementia. 2021. www.alzheimersresearchuk.org/wp‐content/uploads/2021/05/ARUK‐Are‐we‐ready‐to‐deliver‐disease‐modifying‐treatments_25May21.pdf (accessed January 13, 2025)

[38] Duara R , Barker W . Heterogeneity in Alzheimer's disease diagnosis and progression rates: implications for therapeutic trials. Neurotherapeutics. 2022;19(1):8‐25. doi:10.1007/s13311-022-01185-z 35084721 PMC9130395

[39] Tijms BM , Vromen EM , Mjaavatten O , et al. Cerebrospinal fluid proteomics in patients with Alzheimer's disease reveals five molecular subtypes with distinct genetic risk profiles. Nat Aging. 2024;4(1):33‐47. doi:10.1038/s43587-023-00550-7 38195725 PMC10798889

[40] Schneider JA , Arvanitakis Z , Bang W , Bennett DA . Mixed brain pathologies account for most dementia cases in community‐dwelling older persons. Neurology. 2007;69(24):2197‐2204. doi:10.1212/01.wnl.0000271090.28148.24 17568013

[41] Brenowitz WD , Hubbard RA , Keene CD , et al. Mixed neuropathologies and estimated rates of clinical progression in a large autopsy sample. Alzheimers Dement. 2017;13(6):654‐662. doi:10.1016/j.jalz.2016.09.015 27870939 PMC5438283

[42] Barnes LL , Leurgans S , Aggarwal NT , et al. Mixed pathology is more likely in black than white decedents with Alzheimer dementia. Neurology. 2015;85(6):528‐534. doi:10.1212/WNL.0000000000001834 26180136 PMC4540250

[43] Korczyn AD , Grinberg LT . Is Alzheimer disease a disease?. Nat Rev Neurol. 2024;20:245‐251. doi:10.1038/s41582-024-00940-4 38424454 PMC12077607

[44] Alzheimer's network for treatment and diagnostics | ALZ‐NET n.d. https://www.alz‐net.org/(accessed September 9, 2025)

